# Objective Measurement of Listening Device Use and Its Relation to Hearing Acuity

**DOI:** 10.1177/01945998211012274

**Published:** 2021-05-25

**Authors:** Danique E. Paping, Jantien L. Vroegop, Geert Geleijnse, Carlijn M.P. le Clercq, Simone P.C. Koenraads, Marc P. van der Schroeff

**Affiliations:** 1 Department of Otorhinolaryngology–Head and Neck Surgery, Erasmus University Medical Center, Rotterdam, the Netherlands; 2 The Generation R Study Group, Erasmus University Medical Center, Rotterdam, the Netherlands

**Keywords:** personal listening device, smartphone application, hearing, noise, music

## Abstract

**Objectives:**

To examine whether adolescents exceed recommended noise exposure limits when using personal listening devices (PLDs) and to investigate the relationship between objectively measured PLD use and hearing thresholds

**Study Design:**

Cross-sectional study.

**Setting:**

This study was embedded within an ongoing prospective birth cohort study in Rotterdam, the Netherlands. Data were collected from May 2017 to September 2019.

**Methods:**

A smartphone application was developed to measure daily noise exposure from PLDs. Listening habits were monitored among 314 adolescents with a mean age of 13 years 7 months (SD, 5 months), of whom 51.6% were male. Hearing acuity was measured by pure tone audiometry, and tympanometry was performed in both ears.

**Results:**

Within the study group, 2.2% adolescents exceeded the recommended daily noise dose (85 dBA as an 8-hour time-weighted average) among all days when the application was active and 9.9% when among only the listening days. No significant correlation was found between the daily noise dose from PLDs and pure tone thresholds.

**Conclusions:**

The majority of adolescents exhibited listening habits that could be considered safe. As noise-induced hearing loss develops slowly over time, it could be that the effects of PLD use on hearing are not evident yet in this young population with a relatively short duration of PLD use.

Personal listening devices (PLDs) such as smartphones and MP3 players with audio playback function have become increasingly popular. More than 90% of adolescents and young adults use PLDs on a regular basis, with smartphones the most commonly used.^1-3^ The World Health Organization has expressed concern over the risk of hearing loss due to unsafe listening practices among young people.^4^ Although researchers agree that PLDs are capable of producing hazardous output levels,^5-7^ the actual risk of hearing loss due to PLD use remains a subject of debate. Whereas some studies demonstrated that hearing thresholds are significantly elevated in teenagers and young adults who use PLDs,^8-12^ others did not observe an association between PLD use and hearing at the conventional frequencies.^13-15^ Since hearing loss can lead to a number of disabilities and can reduce the quality of life,^16^ prevention is of great importance.

The results of previous described studies are based on self-reports of PLD use, sometimes in combination with physical measurements of preferred listening levels. However, little is known about the validity of these measures. Self-reports are prone to recall bias, and single measurements assume that listeners do not change their exposure levels during the day or week. To date, no studies have evaluated the association between objectively measured PLD use and hearing thresholds. In search of ways to objectively monitor noise exposure, we developed a smartphone application that was able to collect data on listening habits in a large population-based cohort of adolescents. The first aim was to determine daily noise exposure from PLDs and examine whether adolescents exceed recommended exposure limits. The second aim was to investigate the dose-response relationship between daily noise exposure from PLDs and pure tone thresholds.

## Methods

### Study Design and Population

This study was embedded within Generation R, a population-based prospective cohort study from fetal life onward in Rotterdam, the Netherlands. The design and population have been described.^17^ Adolescents aged 12 to 17 years who visited the research center at the Erasmus Medical Center between May 2017 and September 2019 were invited to participate in this substudy (n = 3237). To be eligible to participate, adolescents needed to have a smartphone. The first version of the application was designed only for smartphones running the Android operation. The updated version, available from April 2019 onward, ran on iOS smartphones as well. The study was approved by the Medical Ethical Committee at the Erasmus Medical Center. Written informed consent was obtained from all participants and their parents.

### Smartphone Application

The application was overseen by the departments of communication, security, legal affairs, and data protection of the Erasmus Medical Center. Several rounds of technical development and field testing were conducted before the application was implemented within the Generation R Study. The application was tested 3 times by 10 individuals, after which adjustments to the design and software were made. The application was aimed to record listening habits (ie, frequency, listening time, and listening level). Participants were able to use their own headphones. To log in, a unique username and password were required, provided during participants’ visit at the research center. After logging in, participants received a questionnaire regarding the brand and type of smartphone, volume limit settings, the use of earphones or headphones for listening, and whether they tend to listen to music with 1 or 2 ears. When participants completed the questionnaire, the application started to run constantly in the background to collect data on listening habits. For the specific methodology on how listening habits were recorded, see Paping et al.^18^ Although the application period was intended to be 35 days, participants could delete the application at any time. After 35 days, participants received a notification that the study ended and the application could be deleted. Yet, some participants did not remove the application, resulting in a monitoring period >35 days. Participants were included in the analyses if the time of installation or the time between first and last measurement was between 7 and 40 days.

#### Daily Noise Dose

Consistent with previous studies, the daily noise dose was calculated on the basis of occupational safety standards. The National Institute for Occupational Safety and Health (NIOSH) defines a time-weighted average of 85 A- weighted decibels (dBA) for an 8-hour period as the maximum recommended noise dose per day.^19^ For every 3-dB increase in noise level, the allowable exposure time is reduced by half. Exposures from individual activities in a given day are added to calculate the daily noise dose. The recommended daily noise dose should not be exceeded, as it places an individual at a higher risk of acquired hearing loss.

The data collected by the application were used to calculate participants’ daily noise dose in several stages. First, we converted the listening levels into estimated output levels in dBA with a regression equation from literature. Several studies have evaluated output levels of smartphones, reporting different results.^5,14,20,21^ Therefore, we performed our own measurement with a modified probe test cavity of Otodynamics. A hole was drilled at the bottom of the cavity to tightly fit a Brüel & Kjaer 4189 microphone, and intra-aural earphones were inserted into the cavity (Supplemental Figure S1, available online). For 3 devices, output levels were measured at every intensity control level available with a sample of white noise (Supplemental Figure S2). As the output levels measured were most compatible with the study of Williams et al (Supplemental Figure S3), this regression equation was used to convert listening levels to sound levels.^21^ The equation is presented by

Soundlevel(dBA)=0.5904×volumelevel(%)+38.78

Hereafter, the daily noise doses were calculated with the following formula:

$$D\;=\frac{C_{1}}{T_{1}}+\frac{C_{2}}{T_{2}}+\ldots +\frac{\mathrm{Cn}}{\mathrm{Tn}}\;\times \;100,\mathrm{where}$$

*D* = daily noise dose (%), *C_n_* = total time of exposure at a specified noise level (min), and *T_n_* = exposure time at which the noise for this specified level becomes hazardous (min). The exposure time at which the noise for a specified level becomes hazardous was calculated by

$$\mathrm{Tn}=\;\frac{480}{\;2^{\frac{\left(L-85\right)}{3}}},\mathrm{where}$$

*T_n_* = exposure time at which the noise for this level becomes hazardous (min) and *L* = exposure level (dBA). Last, the mean daily noise dose was calculated for each participant by adding all daily noise doses and dividing by the number of days that the application was active (daily noise dose–all days) or the number of listening days (daily noise dose–listening days).

#### Audiologic Measures

Participants underwent pure tone audiometry and tympanometry in a sound-treated booth meeting the maximum permissible ambient sound pressure levels of ISO standard 8253-1 (International Organization for Standardization). Hearing assessment was performed before participants installed the application. Air conduction thresholds were obtained from 0.5 to 8 kHz via a clinical audiometer (Decos audiology workstation version 210.2.6 with AudioNigma interface) and TDH-39P headphones with MX-41/AR ear cushions. Due to time constraints, no bone conduction thresholds were measured. All thresholds were measured according to the shortened ascending method based on ISO standard 8253-1, which means that thresholds were defined by the intensity level at which the tone was heard in 2 of 3 ascents. The right and left ears were alternately tested first. Tympanometry (Interacoustics AT235h tympanometer with a 226-Hz probe tone) was used to assess middle ear function. Ear canal volume >0.3 mL, compliance >0.25 mL, and middle ear pressure between −100 and 100 daPa were considered normal.^22^

Noise-induced hearing loss is often presented as a notch between 3 and 6 kHz or as high-frequency hearing loss.^23,24^ Therefore, we calculated the pure tone average at 3, 4, and 6 kHz (notch) and 6 and 8 kHz (high frequency). When participants reported listening to the PLD with both ears, we averaged the pure tone average of the right and left ears. Otherwise, the pure tone average of the right or left ear was selected, depending on which the participant predominantly listened with. Participants who had missing or abnormal tympanometry with a pure tone average >15 dB HL (notch or high frequency) were excluded to avoid possible inference of external or middle ear pathology when the relationship between noise exposure from PLDs and hearing thresholds was examined.

#### Demographic Characteristics

Data on demographics were obtained by parental questionnaires. Ethnicity was categorized as Western (Dutch, European, American Western, Asian Western, Oceanian) or non-Western.^17^

#### Statistics

Statistical analyses were performed in IBM SPSS statistics version 24 and R version 3.6.1. Descriptive statistics were used to evaluate participants’ demographic characteristics and listening habits. Spearman correlation coefficients were calculated to evaluate the correlation between daily noise dose and pure tone thresholds, which did not show a normal distribution. A *P* value <.05 was considered statistically significant.

### Results

**Figure 1.**
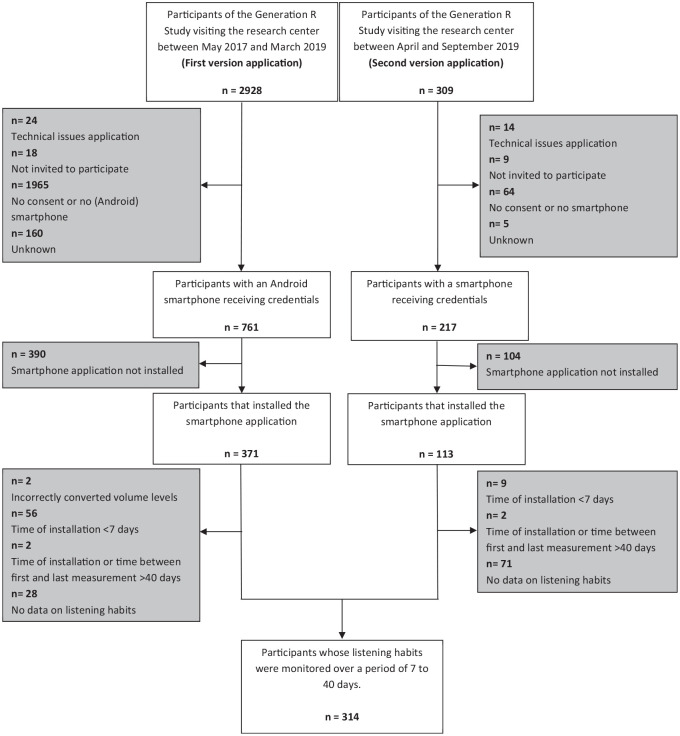
Flowchart of study sample.

Of the 3237 adolescents visiting the research center between May 2016 and September 2019, 978 (30.2%) agreed to participate in this substudy. Of these participants, 484 (49.5%) actually installed and started the application. We excluded participants with a OnePlus smartphone, as listening levels were incorrectly converted (n = 2), and participants with an installation time or a time between first and last measurement of <7 days or >40 days (n = 69). After the exclusion of participants who did not listen to music or watch a video with headphones at least once while the application was active (n = 99), 314 participants were included (**Figure 1**). The demographics of the included participants are presented in **Table 1**. Differences in demographic characteristics between the included and excluded participants are shown in Supplemental Table S1 (available online).

**Table 1.** Demographic Characteristics.^a^

Characteristic	No. (%)
Age, y, mean (SD)	13 y 7 mo (5 mo)
Sex	
Male	162 (51.6)
Female	152 (48.4)
Ethnicity^b^	
Western	231 (73.6)
Non-Western	81 (25.8)
Unknown	2 (0.6)
Educational level^c^	
Lower	64 (20.4)
Intermediate	51 (16.2)
Higher	160 (51.0)
Unknown	39 (12.4)
Household income, €^d^	
<2800	71 (22.6)
≥2800	194 (61.8)
Unknown	49 (15.6)

a Between-group differences were examined via the independent samples *t* test and Pearson chi-square test. Differences were based on participants with complete data regarding the variable.

b Western ethnicity included Dutch, European, American Western (including North American), Asian Western (including Indonesian and Japanese) and Oceanian. Non-Western ethnicity included Turkish, Moroccan, Surinamese, Antillean, Cape Verdean, African, Asian (except Indonesia and Japan), South American, and Central American.

c The educational level of the participant was classified as low (primary education only or preparatory secondary vocational education), middle (senior general secondary education), or high (university preparatory education).

d A net household income was classified as below the national average (<€2800) or equal to or above the national average (≥€2800).

#### Listening Habits

Most participants had a Samsung smartphone (63.7%), followed by Huawei (11.1%) and Motorola (9.2%). Apple was less common (4.1%), since the first version of the application was designed for smartphones running the Android operation only. Earphones were used by 80.3% of the participants and headphones by 19.7%. The majority reported listening to music with both ears (80.6%); 12.1% listened predominantly with the right ear and 7.3% with the left. Listening habits were monitored over a median course of 33 days (interquartile range [IQR], 22.0-35.0). The median number of days listening a week was 2.4 (IQR, 1.0-3.3). The median listening time was 20.2 minutes a day (IQR, 8.8-50.8) when averaged over all days that the application was active and 81.5 minutes (IQR, 46.7-128.0) when calculated over only the listening days. The mean listening level was 55.0% (SD, 17.9%). When the listening habits of Android (n = 301) and Apple (n = 13) users were compared, we observed that Apple users listened less frequently (median, 0.8 vs 2.1 days) and of shorter duration (median, 6.5 vs 21.1 minutes). The listening levels were comparable (mean, 56.6% vs 55.0%).

The majority of participants varied their listening habits during the month (ie, frequency and listening time). No specific pattern was observed. The mean listening time did not differ among days of the week (Supplemental Figure S4, available online). Listening levels changed to a lesser extent.

#### Daily Noise Dose and Pure Tone Audiometry

**Figure 2.**
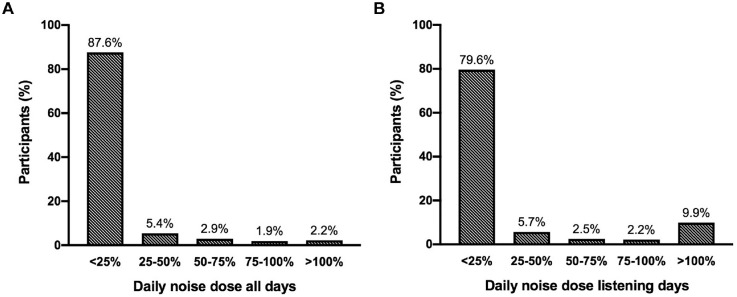
Distribution of participants’ mean daily noise dose averaged over (A) all days when the application was active and (B) only the listening days.

**Figure 3.**
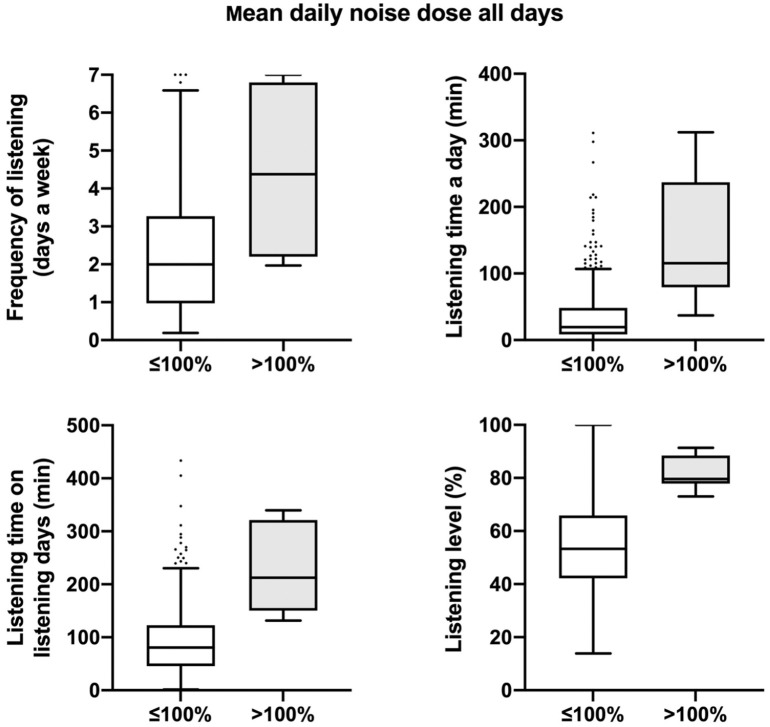
Box plots of participants’ listening habits with mean daily noise dose (≤100% and >100%) averaged over all days when the application was active. Values are presented as median (line), interquartile range (box), 95% CI (error bars), and outliers (circles).

The cohort’s median daily noise dose was 0.7% (IQR, 0.1%-6.3%) for all days and 2.7% (IQR, 0.2%-17.1%) for the listening days. Seven participants (2.2%) exceeded the 100% daily noise dose for all days and 31 (9.9%) for the listening days. The 100% daily noise dose was exceeded at least once during the monitoring period by 28.8% of the participants. **Figure 2** represents the distribution of participants’ mean daily noise dose. In general, participants with a daily noise dose for all days >100% listened more frequently, for a longer period, and at a higher listening level than participants with a daily noise dose ≤100% (**Figure 3**).

**Figure 4.**
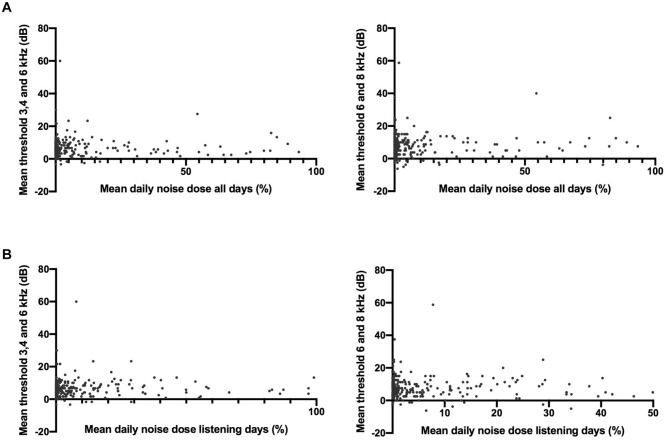
Participants’ mean daily noise dose plotted against the average pure tone threshold at 3, 4, and 6 kHz and 6 and 8 kHz. Two data points are outside the limit of the x-axis.

Of the 314 participants whose listening habits were monitored, 5 (1.6%) were excluded as no pure tone thresholds were obtained and 10 (3.2%) because of missing or abnormal tympanometry in combination with a pure tone average >15 dB HL (notch or high frequency). For the remaining 299 participants, scatterplots were created and Spearman correlation coefficients calculated. No relationship was observed between the daily noise dose and pure tone thresholds (**Figure 4**). Spearman correlation revealed no significant correlations, as demonstrated in **Table 2**. Results did not change when outliers (±3 SD) were eliminated from the analyses (all *P* > .8).

**Table 2.** Correlation Between the Daily Noise Dose and Pure Tone Average Across the Frequencies 3, 4, and 6 kHz and 6 and 8 kHz.

	Spearman correlation coefficient	*P* value
Daily noise dose–all days^a^		
PTA-notch^b^	0.051	.375
PTA-HF^c^	0.000	.998
Daily noise dose–listening days^d^		
PTA-notch	0.068	.242
PTA-HF	0.032	.577

a The mean daily noise dose when averaged over all days when the application was active.

b Pure tone average across the frequencies 3, 4, and 6 kHz (notch).

c Pure tone average across the frequencies 6 and 8 kHz (high frequency).

d The mean daily noise dose when averaged over the listening days.

### Discussion

About 2% of the adolescents in this population-based study were estimated to exceed the NIOSH safety standard for occupational noise^19^ solely by listening to PLDs. An increase to 9.9% was observed when only listening days were taken into account. To our knowledge, just 1 study has measured PLD use with a smartphone application.^25^ The 100% daily noise dose was exceeded by 2.7% of the participants, which is consistent with our findings. Studies based on self-reports to assess daily noise exposure found that 16% to 26% of young people exceed the safety standard when using PLDs.^26,27^ This finding may indicate that participants tend to overestimate their listening habits and that self-reports cannot predict the actual daily noise dose sufficiently. In the present study, we observed that listening habits are highly variable. Self-reports measuring listening habits at a single point in time may not account for this variability. Our research group recently examined the accuracy of self-reported listening habits.^18^ The results showed that the majority of adolescents were not able to estimate their PLD use in a reliable manner. Listening habits were overestimated by a quarter to a half of the participants. We therefore consider an application of added value when assessing PLD use.

Another possible explanation for the lower proportion of adolescents exceeding the recommended daily noise dose in present study is that adolescents who frequently expose themselves to harmful listening levels through PLDs decided not to participate. There was a selection toward a relatively Western and highly educated population. Vogel et al found that adolescents with a lower educational level report relatively more exposure to hazardous music levels.^28^ As a result, the number of young people exceeding safety standards when listening to PLDs may be higher in the general population than observed in our study.

No significant association was found between daily noise exposure from PLDs and hearing thresholds. However, the majority of participants had a relatively low daily noise dose, which made it difficult to study the association. Second, as noise-induced hearing loss develops slowly over years of exposure, it could be that the effects of overexposure to PLDs are not yet evident in this population with a relatively short duration of PLD use. Kim et al found no association between daily PLD use and hearing thresholds, but students who used their PLDs for >5 years had significantly elevated hearing thresholds.^8^

#### Strengths and Limitations

An application was developed for this study to collect real-time data on PLD listening habits. The application was easy to use and required minimal storage capacity and battery. A limitation was that the application could be installed on only a single PLD. If participants used multiple PLDs, the daily noise dose is an underestimation of true value. Meanwhile, it is also possible that the smartphone was used by someone other than the participant or that the earphones or headphones were not worn during a listening session, resulting in an overestimation of the listening habits.

To calculate participants’ daily noise dose, the listening levels registered by the smartphone were converted into estimated output levels via a regression equation.^21^ According to the literature, output levels vary by the type of headphones used and style of music.^5,7,20^ We did not take these factors into account, which could lead to an over- or underestimation of participants’ true daily noise dose. However, we expect this possible bias to be nondifferential. Furthermore, when daily noise dose is calculated, other sources of recreational and occupational noise exposure should be taken into account. We would therefore recommend a lower cutoff for the maximum daily noise dose due to PLDs alone.

In line with previous research, the daily noise dose was calculated on the basis of occupational safety standards. To date, there are hardly any guidelines on recreational noise exposure. As there are authors suggesting that it is not appropriate to simply adopt current occupational exposure limits to recreation settings,^29^ the results should be interpreted with caution.

The current study was conducted as part of the Generation R Study. Hearing acuity was assessed by dedicated research assistants with a small variance, resulting in a relatively homogenous setting. A limitation of this study is that otoscopic examination and bone conduction audiometry were not performed due to time constraints. Since our main interest was sensorineural hearing loss, we decided to include only participants with normal tympanometry in case of elevated thresholds.

Unfortunately, the participation rate was lower than expected. As mentioned previously, there was a selection toward a relatively Western and highly educated population, which may affect the generalizability of our findings. A lack of consent and not having an Android smartphone were the main reasons for exclusion. The lower response rate due to no consent may be caused by the limited time at the research center allocated to inform about the application. We observed that the willingness to participate increased when more information was provided. It is possible that we did not have adequate power to detect an association between PLD use and hearing acuity in present study, especially considering the small sample exceeding the recommended daily noise dose.

The findings of this study revealed several important knowledge gaps, including the impact of long-term PLD use and other sources of recreational noise exposure on young people’s hearing. It would also be of interest to examine which sociodemographic and psychosocial factors are related to unsafe listening habits, for the purpose of prevention.

#### Future Research

The application is currently being developed. An important adjustment is that adolescents using Bluetooth headphones can also participate in the new version of the application. Hearing acuity and listening habits will be repeatedly measured in the future. We aim to examine the change in listening habits with age and the long-term impact of PLD use on hearing.

### Conclusions

The majority of adolescents exhibited safe listening habits according to NIOSH criteria. The degree of noise exposure to PLDs was not associated with hearing acuity. As noise-induced hearing loss develops slowly over time, it could be that the effect of PLD is not evident yet in this young population with a relatively short duration of PLD use. Moreover, it is possible that an association between PLD use and hearing acuity would emerge with an increase in sample size.

## Supplemental Material

sj-docx-5-oto-10.1177_01945998211012274 – Supplemental material for Objective Measurement of Listening Device Use and Its Relation to Hearing Acuity Click here for additional data file. Supplemental material, sj-docx-5-oto-10.1177_01945998211012274 for Objective Measurement of Listening Device Use and Its Relation to Hearing Acuity by Danique E. Paping, Jantien L. Vroegop, Geert Geleijnse, MSc, Carlijn M.P. le Clercq, Simone P.C. Koenraads and Marc P. van der Schroeff in Otolaryngology–Head and Neck Surgery

sj-jpg-1-oto-10.1177_01945998211012274 – Supplemental material for Objective Measurement of Listening Device Use and Its Relation to Hearing Acuity Click here for additional data file. Supplemental material, sj-jpg-1-oto-10.1177_01945998211012274 for Objective Measurement of Listening Device Use and Its Relation to Hearing Acuity by Danique E. Paping, Jantien L. Vroegop, Geert Geleijnse, MSc, Carlijn M.P. le Clercq, Simone P.C. Koenraads and Marc P. van der Schroeff in Otolaryngology–Head and Neck Surgery

sj-jpg-2-oto-10.1177_01945998211012274 – Supplemental material for Objective Measurement of Listening Device Use and Its Relation to Hearing Acuity Click here for additional data file. Supplemental material, sj-jpg-2-oto-10.1177_01945998211012274 for Objective Measurement of Listening Device Use and Its Relation to Hearing Acuity by Danique E. Paping, Jantien L. Vroegop, Geert Geleijnse, MSc, Carlijn M.P. le Clercq, Simone P.C. Koenraads and Marc P. van der Schroeff in Otolaryngology–Head and Neck Surgery

sj-jpg-3-oto-10.1177_01945998211012274 – Supplemental material for Objective Measurement of Listening Device Use and Its Relation to Hearing Acuity Click here for additional data file. Supplemental material, sj-jpg-3-oto-10.1177_01945998211012274 for Objective Measurement of Listening Device Use and Its Relation to Hearing Acuity by Danique E. Paping, Jantien L. Vroegop, Geert Geleijnse, MSc, Carlijn M.P. le Clercq, Simone P.C. Koenraads and Marc P. van der Schroeff in Otolaryngology–Head and Neck Surgery

sj-jpg-4-oto-10.1177_01945998211012274 – Supplemental material for Objective Measurement of Listening Device Use and Its Relation to Hearing Acuity Click here for additional data file. Supplemental material, sj-jpg-4-oto-10.1177_01945998211012274 for Objective Measurement of Listening Device Use and Its Relation to Hearing Acuity by Danique E. Paping, Jantien L. Vroegop, Geert Geleijnse, MSc, Carlijn M.P. le Clercq, Simone P.C. Koenraads and Marc P. van der Schroeff in Otolaryngology–Head and Neck Surgery

## References

[1] Henderson E Testa MA Hartnick C. Prevalence of noise-induced hearing-threshold shifts and hearing loss among US youths. Pediatrics. 2011;127:e39-e46. 21187306 10.1542/peds.2010-0926

[2] Vogel I Brug J Van der Ploeg CPB , et al. Adolescents risky MP3-player listening and its psychosocial correlates. Health Educ Res. 2011;26:254-264. 21321009 10.1093/her/cyq091

[3] Sulaiman AH Husain R Seluakumaran K. Hearing risk among young personal listening device users: effects at high-frequency and extended high-frequency audiogram thresholds. J Int Adv Otol. 2015;11(2):104-109. 26380997 10.5152/iao.2015.699

[4] World Health Organization. 1.1 billion people at risk of hearing loss. Accessed September 2, 2020. http://www.who.int/mediacentre/news/releases/2015/ear-care/en/

[5] Breinbauer HA Anabalón JL Gutierrez D , et al. Output capabilities of personal music players and assessment of preferred listening levels of test subjects: outlining recommendations for preventing music-induced hearing loss. Laryngoscope. 2012;122:2549-2556. 23060148 10.1002/lary.23596

[6] Portnuff CDF Fligor BJ Arehart KH . Teenage use of portable listening devices: a hazard to hearing? J Am Acad Audiol. 2011;22(10):663-677. 22212766 10.3766/jaaa.22.10.5

[7] Shim H Lee S Koo M , et al. Analysis of output levels of an MP3 player: effects of earphone type, music genre, and listening duration. J Audiol Otol. 2018;22:140. 29471612 10.7874/jao.2017.00339 PMC6103496

[8] Kim MG Hong SM Shim HJ , et al. Hearing threshold of Korean adolescents associated with the use of personal music players. Yonsei Med J. 2009;50:771-776. 20046416 10.3349/ymj.2009.50.6.771 PMC2796402

[9] Le Clercq CMP Goedegebure A Jaddoe VWV , et al. Association between portable music player use and hearing loss among children of school age in the Netherlands. JAMA Otolaryngol Head Neck Surg. 2018;144:668-675. 29902307 10.1001/jamaoto.2018.0646 PMC6143000

[10] Peng J-H Tao Z-Z Huang Z-W. Risk of damage to hearing from personal listening devices in young adults. J Otolaryngol. 2007;36(3):181-185. 17711774

[11] Widén SE Båsjö S Möller C , et al. Headphone listening habits and hearing thresholds in Swedish adolescents. Noise Health. 2017;19:125. 28615542 10.4103/nah.NAH_65_16 PMC5501022

[12] Hussain T Chou C Zettner E , et al. Early indication of noise-induced hearing loss in young adult users of personal listening devices. Ann Otol Rhinol Laryngol. 2018;127:703-709. 30056742 10.1177/0003489418790284

[13] Mostafapour SP Lahargoue K Gates GA. Noise-induced hearing loss in young adults: the role of personal listening devices and other sources of leisure noise. Laryngoscope. 1998;108:1832-1839. 9851500 10.1097/00005537-199812000-00013

[14] Kumar A Mathew K Alexander S , et al. Output sound pressure levels of personal music systems and their effect on hearing. Noise Health. 2009;11:132. 19602765 10.4103/1463-1741.53357

[15] Sulaiman AH Husain R Seluakumaran K. Evaluation of early hearing damage in personal listening device users using extended high-frequency audiometry and otoacoustic emissions. Eur Arch Otorhinolaryngol. 2014;271:1463-1470. 23812554 10.1007/s00405-013-2612-z

[16] Arlinger S. Negative consequences of uncorrected hearing loss—a review. Int J Audiol. 2003;42:2S17-2S20. 12918624

[17] Kooijman MN Kruithof CJ van Duijn CM , et al. The Generation R Study: design and cohort update 2017. Eur J Epidemiol. 2016;31:1243-1264. 28070760 10.1007/s10654-016-0224-9 PMC5233749

[18] Paping DE Vroegop JL Koenraads SP , et al. A smartphone application to objectively monitor music listening habits in adolescents. J Otolaryngol Head Neck Surg. 2021;50:1-10. 34384502 10.1186/s40463-021-00532-y PMC8361637

[19] National Institute for Occupational Safety and Health. Criteria for a recommended standard. Published June 1998. https://www.cdc.gov/niosh/docs/98-126/pdfs/98-126.pdf

[20] Kim G Han W. Sound pressure levels generated at risk volume steps of portable listening devices: types of smartphone and genres of music. BMC Public Health. 2018;18:481. 29712550 10.1186/s12889-018-5399-4 PMC5928569

[21] Williams W Purnell J Parnell J , et al. The statistical distribution of expected noise level output from commonly available personal stereo players. Acoustics Australia. 2010;38:119-122.

[22] Jerger J. Clinical experience with impedance audiometry. Arch Otolaryngol. 1970;92:311-324. 5455571 10.1001/archotol.1970.04310040005002

[23] Le Clercq CMP Van Ingen G Ruytjens L , et al. Music-induced hearing loss in children, adolescents, and young adults: a systematic review and meta-analysis. Otol Neurotol. 2016;37:1208-1216. 27466893 10.1097/MAO.0000000000001163

[24] McBride DI Williams S. Audiometric notch as a sign of noise induced hearing loss. Occup Environ Med. 2001;58:46-51. 11119634 10.1136/oem.58.1.46 PMC1740031

[25] Kaplan-Neeman R Muchnik C Amir N. Listening to music with personal listening devices: monitoring the noise dose using a smartphone application. Int J Audiol. 2017;56:400-407. 28281836 10.1080/14992027.2017.1297541

[26] Lee GJC Lim MY Kuan AYW , et al. The music listening preferences and habits of youths in Singapore and its relation to leisure noise-induced hearing loss. Singapore Med J. 2014;55:72. 24570315 10.11622/smedj.2014018 PMC4291932

[27] Muchnik C Amir N Shabtai E , et al. Preferred listening levels of personal listening devices in young teenagers: self reports and physical measurements. Int J Audiol. 2012;51:287-293. 22122401 10.3109/14992027.2011.631590

[28] Vogel I Verschuure H van der Ploeg CP , et al. Estimating adolescent risk for hearing loss based on data from a large school-based survey. Am J Public Health. 2010;100:1095-1100. 20395587 10.2105/AJPH.2009.168690 PMC2866607

[29] Roberts B Neitzel RL. Noise exposure limit for children in recreational settings: review of available evidence. J Acoust Soc Am. 2019;146:3922-3933. 31795717 10.1121/1.5132540

