# Supplementary material for: Objective Measurement of Listening Device Use and Its Relation to Hearing Acuity
Source: Otolaryngol Head Neck Surg. 2021 May 25;166(3):515–22. doi: 10.1177/01945998211012274 (PMC8892050; doi:10.1177/01945998211012274)
Supplement: sj-docx-5-oto-10.1177_01945998211012274 – Supplemental material for Objective Measurement of Listening Device Use and Its Relation to Hearing Acuity [file sj-docx-5-oto-10.1177_01945998211012274.docx]

**SUPPLEMENTAL DATA FILES**

**Supplemental Table 1.**

 **Included Excluded p-value of the difference^a^**
**Characteristic** n= 314 n= 2923
Age (yr), mean (SD) 13 y 7 mo (5 mo) 13 y 8 mo (5 mo) 0.004
Gender, No. (%)
 Male 162 (51.6) 1413 (48.3) 0.273
 Female 152 (48.4) 1510 (51.7)
Ethnicity**^b^**, No. (%)
 Western 231 (73.6) 1992 (68.1) 0.044
 Non-Western 81 (25.8) 864 (29.6)
 Unknown 2 (0.6) 67 (2.3)
Educational level participant**^c^**, No. (%)
 Lower 64 (20.4) 574 (19.6) 0.015
 Intermediate 51 (16.2) 492 (16.8)
 Higher 160 (51.0) 1291 (44.2)
 Unknown 39 (12.4) 567 (19.4)
Household income**^d^**, No. (%)
 <€2800 71 (22.6) 613 (21.0) 0.017
 ≥€2800 194 (61.8) 1649 (56.4)
 Unknown 49 (15.6) 661 (22.6)

No.= number, SD= standard deviation, yr= year, mo= month
^a^ Between- group differences were examined using the independent samples t-test and Pearson Chi-square test. Differences were calculated based on participants with complete data regarding the variable
^b^ Western ethnicity included Dutch, European, American Western (including North American), Asian Western (including Indonesian and Japanese) and Oceanian. Non-Western ethnicity included Turkish, Moroccan, Surinamese, Antillean, Cape Verdean, African, Asian (except Indonesia and Japan) and South American and Central American.
^c^ The educational level of the participant was classified as low (primary education only or preparatory secondary vocational education), middle (senior general secondary education), or high (university preparatory education)

^d^ A net household income was classified as below the national average ( <€2800), or equal to or above the national average ( ≥€2800).
