# Supplementary figures and images for: Objective Measurement of Listening Device Use and Its Relation to Hearing Acuity
Source: Otolaryngol Head Neck Surg. 2021 May 25;166(3):515–22. doi: 10.1177/01945998211012274 (PMC8892050; doi:10.1177/01945998211012274)

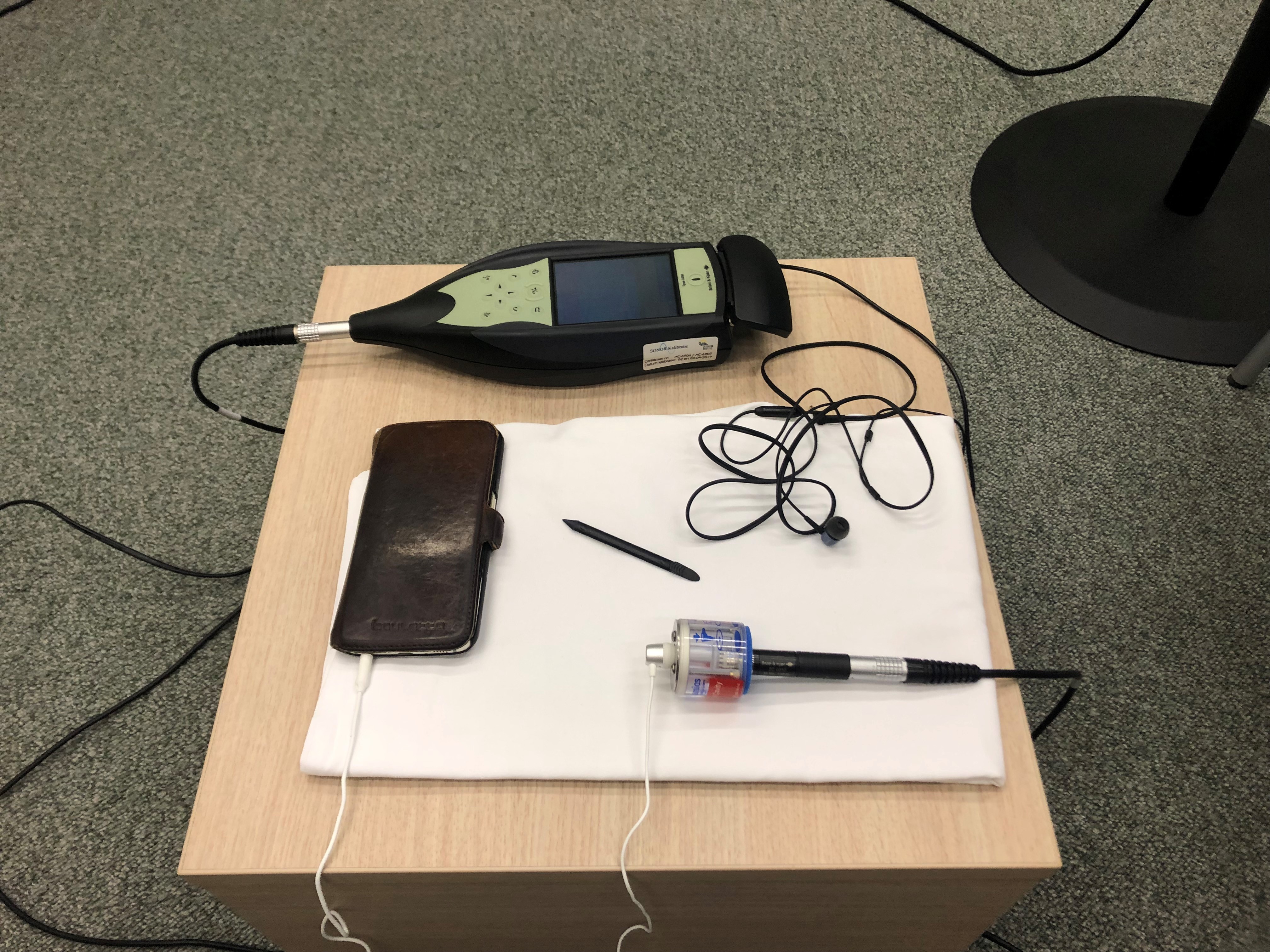

Supplement: sj-jpg-1-oto-10.1177_01945998211012274 – Supplemental material for Objective Measurement of Listening Device Use and Its Relation to Hearing Acuity [file sj-jpg-1-oto-10.1177_01945998211012274.jpg]

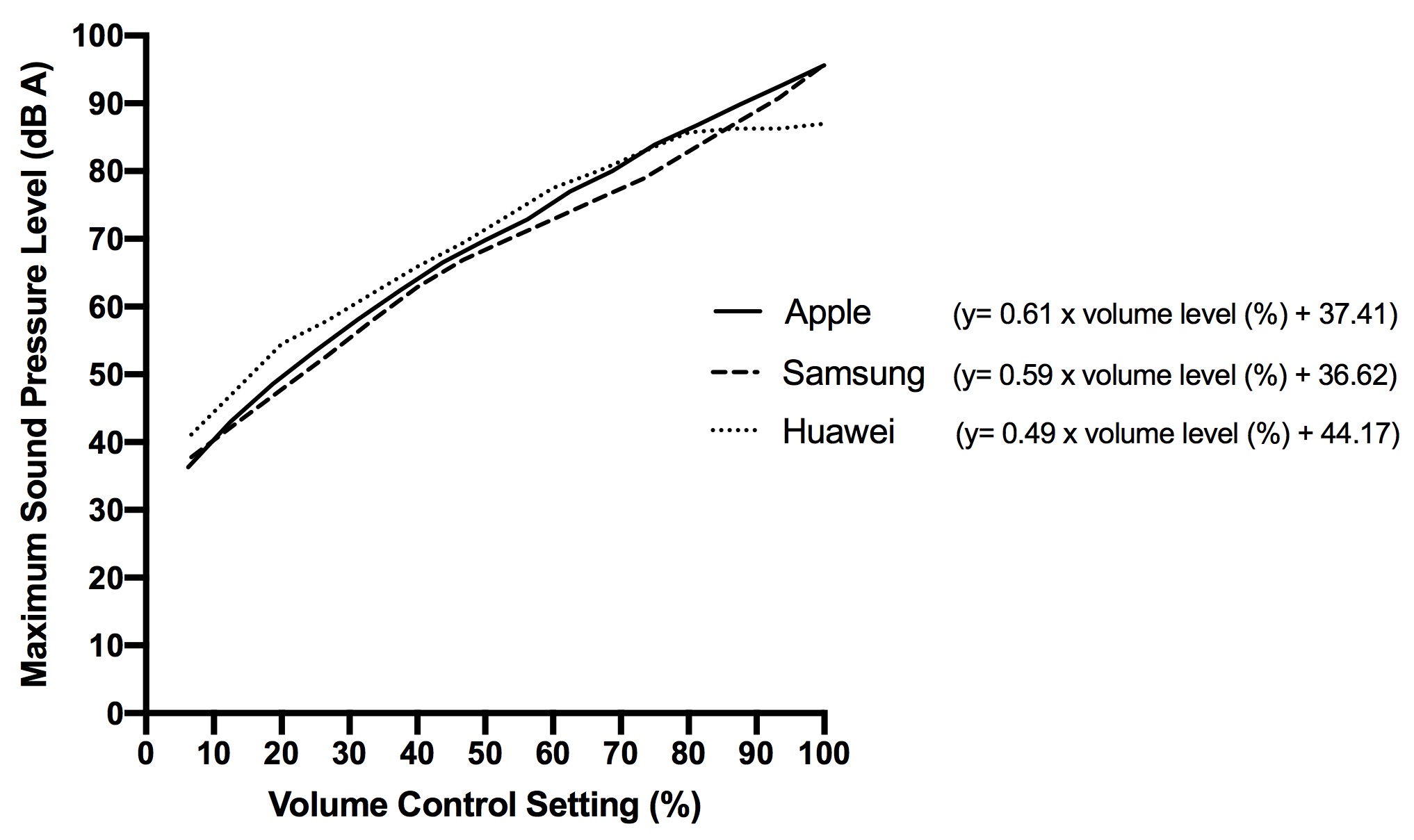

Supplement: sj-jpg-2-oto-10.1177_01945998211012274 – Supplemental material for Objective Measurement of Listening Device Use and Its Relation to Hearing Acuity [file sj-jpg-2-oto-10.1177_01945998211012274.jpg]

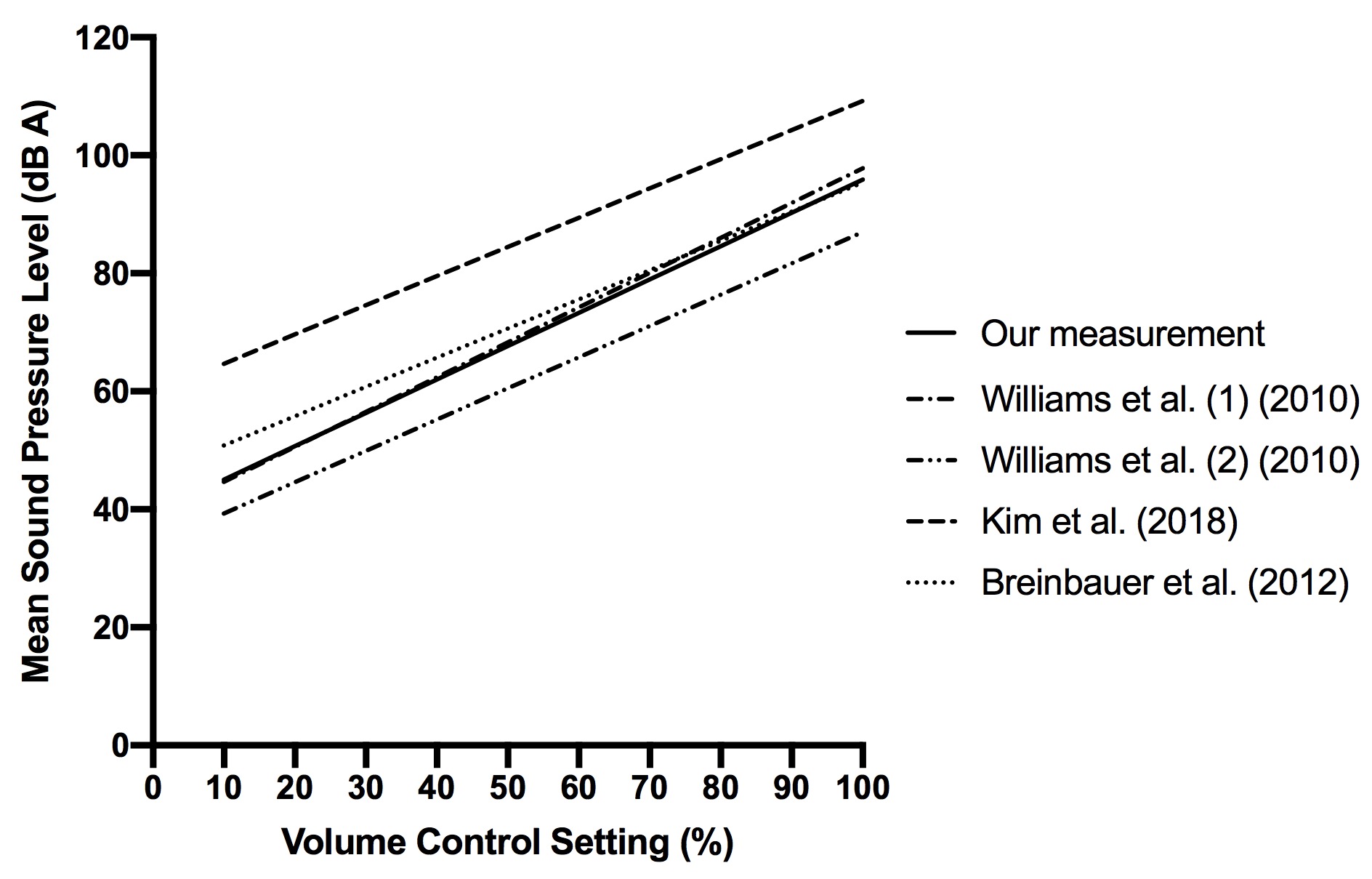

Supplement: sj-jpg-3-oto-10.1177_01945998211012274 – Supplemental material for Objective Measurement of Listening Device Use and Its Relation to Hearing Acuity [file sj-jpg-3-oto-10.1177_01945998211012274.jpg]

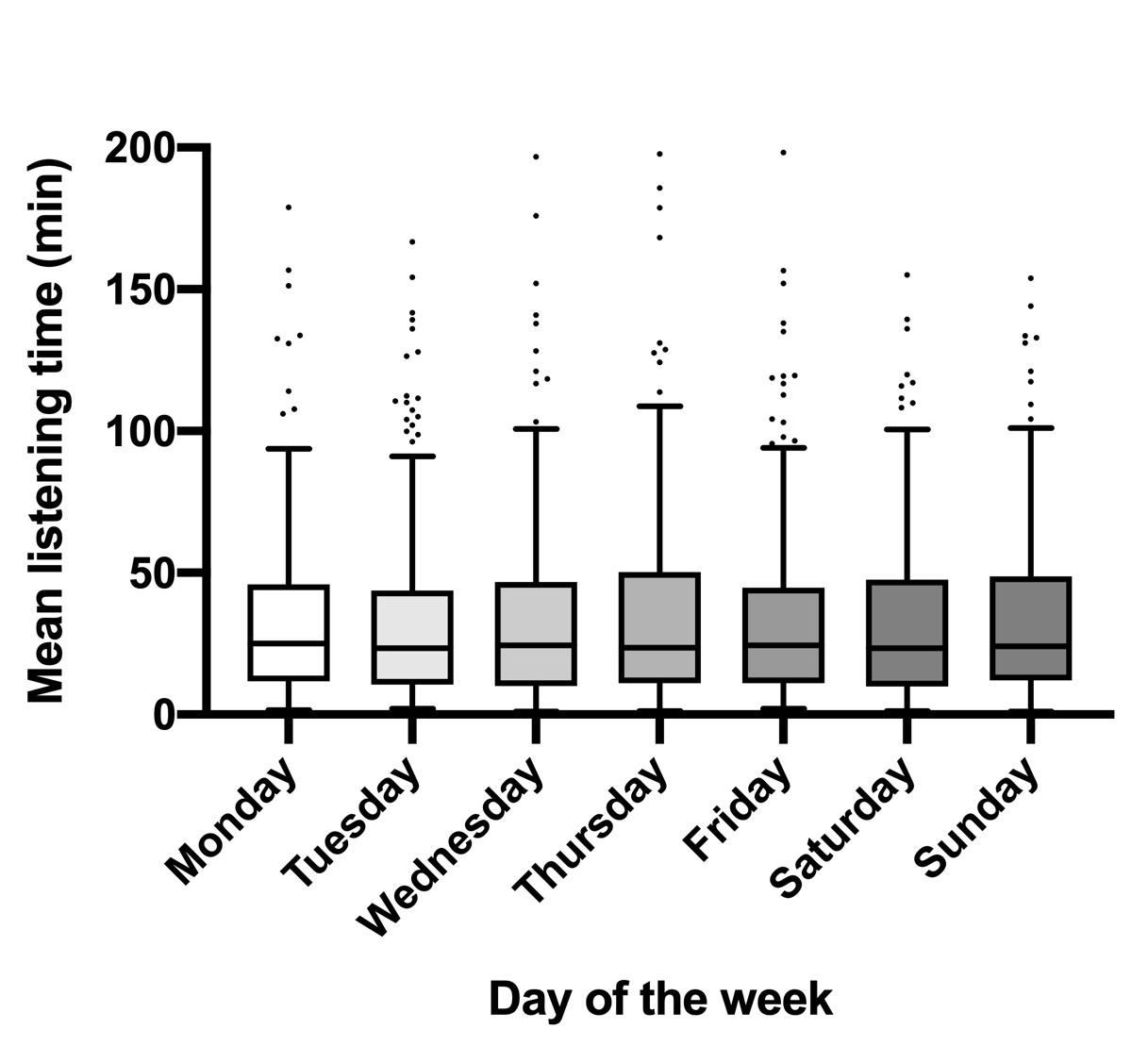

Supplement: sj-jpg-4-oto-10.1177_01945998211012274 – Supplemental material for Objective Measurement of Listening Device Use and Its Relation to Hearing Acuity [file sj-jpg-4-oto-10.1177_01945998211012274.jpg]
